# Role of curcumin on beta-amyloid protein, tau protein, and biochemical and oxidative changes in streptozotocin-induced diabetic rats

**DOI:** 10.1007/s00210-024-03231-3

**Published:** 2024-06-26

**Authors:** Mustafa Ermiş, Gülay Çiftci

**Affiliations:** 1 Erciyes University Experimental Research Application and Research Center, University of Erciyes, Kayseri, Turkey; 2 Department of Veterinary Biochemistry, Faculty of Veterinary Medicine, University of Ondokuz Mayıs, 55220 Atakum, Samsun, Turkey

**Keywords:** Beta amyloid protein, Curcumin, Diabetes, Oxidative stress

## Abstract

Diabetes is one of the most common endocrine metabolic diseases and is associated with the accumulation of beta-amyloid plaques in the brain. Amyloid beta (Aβ) and abnormal tau proteins are effective in the development of Alzheimer’s disease. The aim of this study is to investigate the therapeutic and protective effects of curcumin on beta-amyloid (Aβ) accumulation and tau protein expression levels, as well as biochemical and oxidative changes in streptozotocin-induced diabetes in rats. The study comprised five groups, each consisting of eight rats: control, diabetic, curcumin, curcumin during diabetic induction, and curcumin post-diabetic induction. Groups 2 and 4 were administered a single dose of 45 mg/kg streptozotocin on day 1, while group 5 received it on day 28. Curcumin was orally administered via gavage at a dose of 100 mg/kg/day for 35 days to the third, fourth, and fifth groups. At the end of the trial (day 35), blood sugar levels and insulin resistance were similar between the control and curcumin-treated groups but significantly higher in the diabetic groups (*P* < 0.05). The protective effect of curcumin is tested during induction and active diabetes. The results indicated that diabetic rats displayed increased levels of Aβ, tau protein, and total oxidant capacity (TOS) compared to the curcumin-treated groups. Additionally, the total antioxidant capacity (TAS) levels were lower in the diabetic rats (*P* < 0.05). Aβ protein levels are lower in both the serum and brain of rats with active diabetes and treated with curcumin compared to control rats (*P* > 0.05). In addition, serum TAS levels were higher in rats treated with curcumin following the induction of diabetes than pre-induction of diabetes (*P* > 0.05). The TOS levels in the serum were higher in the rats treated with curcumin during active diabetes compared to the rats treated prior to the induction of diabetes (*P* < 0.05). However, no significant difference was observed in the brain. The above results show that curcumin has an effect on reducing oxidative stress caused by diabetes and increasing antioxidant activity.

## Introduction

Diabetes mellitus is one of the most prevalent endocrine metabolic diseases. Diabetes is driven by decreased response to insulin (insulin resistance) or insufficient insulin production and diagnosed by hyperglycemia. Hyperglycemia increases the accumulation of beta-amyloid plaques in the brain (Chao et al. 2016). In addition, it exacerbates oxidative stress, neuroinflammation, and mitochondrial dysfunction. As a result, it disrupts neuronal integrity and leads to neurodegeneration (Lee et al. 2018). Studies with streptozotocin (STZ)-induced rat model of diabetes show that diabetes leads to oxidative stress, harms pancreatic beta cells, and causes hyperglycemia in rats. Oxidative stress may lead to an excess production of oxygen-free radical precursors and/or a reduction in an effectiveness of antioxidant system (Baynes 1991).

Alzheimer’s disease (AD), also known as “type 3 diabetes mellitus (DM),” is associated with impaired insulin signaling observed in this condition. Both diseases are affected by aging, oxidative stress, inflammation, disrupted energy metabolism, insulin insufficiency, or insulin resistance (Silveira et al. 2019). AD is a progressive, neurodegenerative disease characterized by extracellular amyloid beta (Aβ) protein deposition and intracellular neurofibrillary tangles of hyperphosphorylated tau protein (Kurkinen et al. 2023). Insulin resistance increases the hyperphosphorylation of tau protein, expression of amyloid precursor protein, and the formation and accumulation of beta-amyloid plaques (Hamze et al. 2022). Oxidative stress exacerbates neuroinflammation and mitochondrial dysfunction leading to disruption of neuronal integrity and neurodegeneration (Lee et al. 2018). Curcumin possesses a diverse array of biological properties such as antioxidant, anti-adhesion, anticarcinogenic, antimutagenic, and anti-inflammatory effects (Farooqui and Farooqui 2019; Stohs et al. 2020). Among the manifold pharmacological effects, the neuroprotective effects of curcumin have garnered increasing attention. These neuroprotective effects may be linked to the anti-amyloid, and antioxidant properties of curcumin.

Curcumin is a natural compound with a polyphenolic structure. This bioactive ingredient has been proven to have a wide range of functional and biological activities. In addition to the use of aromatic and natural coloring additives in food products, it is also used as a natural antioxidant (Rafiee et al. 2019). Due to its low cost and the absence of any side effects, this antioxidant is used increasinly day by day. It has been shown that it has a high capacity to remove the common oxidative molecules such as H_2_O_2_, HO•, and ROO•. For example, it can be effectively used as an antioxidant to provide protection against ROS in the cytoplasm of cells. The aim of the study was to investigate the therapeutic and protective effects of curcumin application on beta amyloid (Aβ) levels, tau protein expression, and biochemical and oxidative changes in rats with streptozotocin-induced diabetes.

## Materials and methods

### Research site and animal material

Forty healthy male Sprague Dawley rats, age 9–10 weeks, weighing 265 ± 10 g were used. During the study, clinically healthy rats were fed ad libitum and housed at a room temperature of 22 ± 2 °C, a humidity of 60%, and a 12/12-h light/dark environment. During the experiments, the rates were divided into groups according to age range.

### Animal study design

In this study, five groups including eight rats were formed.

- Group 1 (control group): The control group rats were fed with standard rat food for 5 weeks. On the first day of the study, only 0.5 ml of 0.1 M citrate phosphate buffer was injected via intraperitoneally (i.p.) route.
- Group 2 (diabetic group): This group was fed with standard rat food. Diabetes group consisted of eight rats and were fed with standard food and water throughout the 5-week trial. The animals in this group were given a single dose of 45 mg/kg streptozotocin (Cayman) in 0.1 M citrate phosphate buffer pH 4.5 i.p. to induce diabetes on the first day of the study. On the third day of the study, blood glucose levels were measured in samples taken from the tail vein of the rats using a glucometer (Accu-chek Performa, Roche). The fasting blood glucose levels were measured by dripping blood into the strip. Animals with a fasting blood glucose level of 250 mg/dL and above were considered diabetic and the study continued (Ali and Agha 2009).
- Group 3 (curcumin): In this group of eight rats, in addition to being fed with standard food for 5 weeks, curcumin was given by oral gavage at 100 mg/kg/day in 1 mL of drinking water for 5 weeks (Kanter et al. 2013).
- Group 4 (diabetes + curcumin group; therapeutic effectiveness): Diabetes + curcumin group consisted of eight rats and were fed with standard food and water throughout the 5-week trial. The animals in this group were injected intrapetitoneally with a single dose of 45 mg/kg streptozotocin (Cayman) in 0.1 M citrate phosphate buffer pH 4.5 on the first day of the study to initiate diabetes. On the third day of the study, fasting blood glucose levels were measured in samples taken from the tail vein of the rats using a glucometer (Accu-chek Performa, Roche) (Ali and Agha 2009). Animals with a fasting blood glucose level of 250 mg/dL and above were considered diabetic and the study was continued (Zafar et al. 2009). As of the first day of the study, curcumin was administered by oral gavage at a dose of 100 mg/kg/day in 1 mL of drinking water for 5 weeks (Kanter et al. 2013).
- Group 5 (curcumin + diabetes group; protective effectiveness): In this group of eight rats, 100 mg/kg/day of curcumin was orally administered in 1 mL of drinking water for 5 weeks, beginning on the first day of the study. On the 28th day of the study, a single dose of 45 mg/kg streptozotocin was dissolved in 0.1 M citrate phosphate buffer and administered intraperitoneally (Ali and Agha 2009). On the 31st day of the study, fasting blood glucose values were measured as described above. Animals with a fasting blood glucose level of 250 mg/dL and above were considered diabetic and the study was continued.

### Collecting blood and generating serum samples

After 12 h of fasting followed by general anesthesia with xylazine (10 mg/kg body weight intraperitoneal) and ketamine (60 mg ketamine hydrachloride/kg body weight, intraperitoneal), the abdomen and chest areas of rats from different age groups were opened, and blood was drawn directly from their hearts. On this part of the study, sacrification/euthanasia was performed by cervical dislocation method under anesthesia/tranquilizer.The skull of each rat was opened and the brain tissue was removed (Mishra and Flora 2008). Brain tissue was weighed and stored at − 20 °C until biochemical tests were performed.

The blood samples were kept in the laboratory for 20 min and coagulated. Then, they were centrifuged at + 4 °C for 10 min at 1550 × *g*, and their serums were removed and divided into aliquots. Serums were stored at − 20 °C until analysis.

### Measuring the level of some biochemical parameters in serum

Serum samples were used to determine total cholesterol (TC), glucose (Glc), uric acid (UA), aspartate transaminase (AST), alanine aminotransferase (ALT), total protein (TP), albumin (Alb), globulin (Glo), urea, and creatinine levels spectrophotometrically using an autoanalyzer (Biosistem A25, Spain) equipped with the Biosistem kit (Barbour and Davisdon 1988).

### Preparation of brain tissue homogenate for the determination of total beta amyloid protein, tau protein, and insulin level by ELISA

For the preparation of brain tissue homogenization, the tissue was weighed after washing with ice-cold phosphate buffered saline (PBS) (0.01 mol/L, pH 7.0–7.2) before homogenization. After the tissue was cut into small pieces, homogenization was performed with 5–10 mL of PBS in a homogenizer in ice. Then, the cell membrane was disrupted by once or twice with an ultrasonicator in ice, and this is followed by two freeze–thaw cycle for better fragmentation of the cell. The homogenate was centrifuged at 3000 × *g*, 4 °C for 5 min, and the supernatant was divided into aliquots and stored at − 20 °C until analysis. Enzyme-linked ımmunosorbent assay (ELISA) test was performed using supernatants. For this test, appropriate levels of total beta-amyloid protein, tau protein, and insulin in rat-specific brain tissue extracts were measured by ELISA according to the protocol provided with the ELISA test kit, and the results were read using an ELISA reader (Tecan, Crailsheim, Austria) and calculated using Magellan software v1.1.

Total beta amyloid protein (SUNRED Elisa kit, cat no. 201–11-4073), tau protein (SUNRED Elisa kit, cat no. 201–11-4633), and insulin (AFG Bioscience Elisa kit, cat no.EK720161) levels in brain tissue and serum were determined by ELISA method. The ELISAs were performed according to the manufacturer’s instructions, and the absorbances (as OD values) of the ELISA plates were measured by ELISA reader.

The insulin resistance was calculated with the formula of homeostasis model of assesment-insulin resistance (HOMA-IR) as described (Bonora et al. 2000).

### Determination of total antioxidant level (TAS) and total oxidant level (TOS) in serum and all brain tissue

Total antioxidant capacity (TAS) and total oxidant capacity (TOS) in brain tissue supernatants and serum were determined according to the TAS and TOS kits of Rel Assay Diagnostics (Rel Assay®, Diagnostics kits, Mega Tıp, Gaziantep, Turkey). The change in the amount of absorption was measured by spectrophotometric methods. Tissue TAS level was determined by Erel (Erel 2005). This assay relies on the ability of antioxidants in the sample to inhibit oxidation of ABTS (2,2′-azino-di-3-ethylbenz-thiazoline sulfonate) into ABTS^+^ by a peroxidase metmyoglobin. The TAS level was expressed as mmol Trolox equivalent/L (mmol Trolox equiv/L). The tissue TOS level was measured by Erel (Erel 2004). TOS method relied on the oxidation of ferrous ions into ferric ions in the presence of various oxidative species in an acidic medium. Ferric ion concentrations were measured by xylenol orange. Results were expressed as µmol H_2_O_2_ equivalent/L (µmol H_2_O_2_ equiv/L). The TOS/TAS ratio was defined as oxidative stress index (OSI).

### Statistical analyses

The results of the study were analyzed using Statistical Package for Social Sciences, version 23 software. The test results were shown as mean ± standard deviation values in the one-way analysis of variance (ANOVA) test and Duncan’s multiple range-test. A value of *P* < 0.05 was considered statistically significant. Correlations between the variables were evaluated using a Pearson correlation analysis.

## Results

During the experiment, the weights of the rats were regularly determined on a weekly basis. The weekly changes in body weights of the rats in each group are presented in Table 1. At the beginning of the study, the mean body weight of the rats was approximately 265 ± 10 g. At the end of the study (at week 5), body weight changes were determined as 322.1 ± 11.6, 280.5 ± 11.2, 289.2 ± 3.4, 223.6 ± 11.9, 264.1 ± 6 g in groups 1, 2, 3, 4, and 5, respectively. On the 35th day, it was observed that the body weight gain was the highest in the control (group 1) group, and the least in group 4 (*P* < 0.05).

**Table 1:** Body weight (g) changes of the rats in the groups on the 1st, 7th, 14th, 28th, and 35th days

	Group 1	Group 2	Group 3	Group 4	Group 5
1st day	270.7 ± 8.1	265.8 ± 5.3	275.6 ± 10	259.2 ± 4.2	272.6 ± 5.4
7th day	287.3 ± 7.5^a^	260.37 ± 5.4^b^	269.5 ± 6.4^ab^	232 ± 7.3^c^	278.3 ± 6.3^ab^
14th day	297 ± 7.5^a^	264.2 ± 7.4^b^	272.7 ± 5^b^	227.5 ± 7.6^c^	280.5 ± 6.9^ab^
21st. day	309 ± 8.79^a^	271.2 ± 8.3^b^	282.1 ± 4.7^b^	229.8 ± 8.8^c^	294.2 ± 8.1^ab^
28th day	315.8 ± 10^a^	275.2 ± 9.2^b^	281.8 ± 2.6^b^	228.6 ± 10.8^c^	290 ± 8.8^b^
35th day	322.8 ± 11.6^a^	280.5 ± 11.2^b^	289.2 ± 3.4^b^	223.6 ± 11.9^c^	264.1 ± 6^b^

^a,^^b,c^The difference between the groups indicated with different letters in the same column is significant (*P* < 0.05)

Group 1 (control), group 2 (diabetes), group 3 (curcumin), group 4 (diabetes—curcumin), group 5 (curcumin—diabetes)

Throughout the experiment, blood glucose levels of the rats were regularly monitored every week. It was observed that blood glucose levels in groups 2 and 4, which had diabetes, were significantly higher (*P* < 0.05) compared to the control group (group 1), and remained at similar levels in the other groups until the 21st day of the study (Table 2). On the 28th day of the study, blood glucose levels of group 5 were significantly lower than the control group (group 1), at 64.12 (mg/dL), but by the end of the study, they were determined to be significantly higher at 510 (mg/dL) (*P* < 0.05).

**Table 2:** Changes in blood glucose level (mg/dL) of the rats in the groups on the 1st, 7th, 14th, 21st, 28th, and 35th days

	Group 1	Group 2	Group 3	Group 4	Group 5
1st day	107 ± 3.3^a^	389.2 ± 31.3^b^	108.6 ± 3.2^a^	407.5 ± 29.5^b^	120.3 ± 3.6^a^
7th day	108.6 ± 3^a^	366.5 ± 35.7^b^	104.8 ± 12^a^	478.6 ± 38.9^b^	114.7 ± 3.2^a^
14th day	109.7 ± 4.8^a^	428.1 ± 28.1^b^	108.7 ± 3.5^a^	476.1 ± 31.5^b^	111 ± 3.8^a^
21st day	103.7 ± 2^a^	435.9 ± 39.7^b^	103.3 ± 3.8^a^	517.3 ± 27.3^b^	113.8 ± 2.4^a^
28th day	112 ± 8.7^a^	462.9 ± 28.6^b^	106.6 ± 2.7^a^	532.4 ± 29^b^	64.1 ± 10.9^a^
35th day	105.6 ± 2^a^	512.6 ± 26.3^b^	104.1 ± 2.1^a^	511.5 ± 29.8^b^	510 ± 23.6^b^

^a,^^b,c^The difference between the groups indicated with different letters in the same column is significant (*P* < 0.05)

### Levels of some routine biochemical parameters in serum

All measurements were performed in blood samples taken at the end of the experiment (35 days). The control group consisted of group 1 (control), group 2 (diabetes), and group 3 (curcumin) administered for therapeutic purposes, while group 4 (therapeutic efficacy) and group 5 (protective efficacy) were given. Aspartate transaminase (AST), alanine transaminase (ALT), total cholesterol (TC), glucose (Glc), and uric acid (UA) levels in serum were presented in Table 3, total protein (TP), albumin (Alb), and globulin (Glo) levels of the groups are presented in Table 4, and urea and creatinine levels were presented in Table 5 (mean ± SE).

**Table 3:** Some routine biochemical parameters in serum

	Group 1	Group 2	Group 3	Group 4	Group 5
AST (U/L)	87.7 ± 8.1^a^	171.1 ± 35.5^b^	75.8 ± 4.3^a^	164.8 ± 24^b^	229.2 ± 26.1^b^
ALT (U/L)	48.87 ± 2.9^a^	153.75 ± 10.1^b^	55.87 ± 3.1^a^	135.25 ± 28.6^b^	154.75 ± 22.5^b^
TC	65.5 ± 1.1^a^	75.25 ± 3.1^ab^	64.62 ± 6.8^a^	88.5 ± 6.7^b^	126.37 ± 13.4
Glc (mg/dL)	105.62 ± 2^a^	527.65 ± 32.6^b^	104.12 ± 2.1^a^	515.5 ± 33.6^bc^	510 ± 23.6^c^
UA (mg/dL)	1.1 ± 0.1^a^	1.5 ± 0.1^ab^	1.3 ± 0.1^ab^	1.9 ± 0.3^b^	1.38 ± 0.2^ab^
HOMA-IR	2.38	10.59	2.2	11.3	10.35

^a,^^b,c^The difference between the groups shown with different letters on the same line is significant (*P* < 0.05)

The AST level in the serum was found to be significantly higher in groups 2, 4, and 5 with diabetes compared to the control group (group 1) (*P* < 0.05). Its level was the same level in the other groups and not statistically significant (*P* > 0.05). ALT level in serum was found to be significantly higher in groups 2, 4, and 5 with diabetes compared to other groups (groups 1 and 3) (*P* < 0.05). Its level was at the same in the other groups and was not statistically significant (*P* > 0.05).

TC level in the serum was increased in group 5, which developed diabetes at the 4th week, compared to the control groups (*P* < 0.05), while it was at the same level in the other groups, and this was not statistically significant (*P* > 0.05). In groups 2, 4, and 5, which had diabetes, blood glucose levels were found to be significantly higher than the other groups (groups 1 and 3) (*P* < 0.05).

The serum uric acid (UA) level of group 4 was significantly higher than that of the diabetic control group (group 1) (*P* < 0.05). It was determined that there was an increase in the other groups (groups 2, 3, 4, and 5) compared to the control group (group 1), but this was not statistically significant (*P* > 0.05) (Table 3).

**Table 4:** Total protein (TP), albumin (Alb), and globulin (Glo) levels of the groups

	TP (g/L)	Alb (g/L)	Glo (g/L)	Alb/Glo
Groups 1	56.11 ± 1.25^ab^	28.68 ± 0.67^a^	27.42 ± 1^a^	1.04 ± 0.04^a^
Groups 2	53 ± 1.3^ac^	26.7 ± 1.1^a^	26.3 ± 0.88^a^	1.03 ± 0.05^a^
Groups 3	58.7 ± 0.53^b^	29.08 ± 0.29^a^	29.61 ± 0.7^a^	0.98 ± 0.03^a^
Groups 4	55.4 ± 1.48^ab^	26.97 ± 0.78^a^	28.42 ± 0.97^a^	0.95 ± 0.03^a^
Groups 5	50.56 ± 1.49^c^	32.67 ± 1.05^b^	17.87 ± 2.1^b^	2 ± 0.25^b^

^a,^^b,c^Differences between groups indicated with different letters in the same column are significant (*P* < 0.05)

**Table 5:** Urea and creatinine levels in serum

	Urea (mg/dL)	Creatinine (mg/dL)
Group 1	36 ± 1.8^ab^	0.2 ± 0.02^ab^
Group 2	46 ± 5.8^bc^	0.22 ± 0.01^b^
Group 3	36.8 ± 2.4^abc^	0.2 ± 0.02^ab^
Group 4	46.8 ± 3.4^c^	0.23 ± 0.01^b^
Group 5	30.6 ± 1.8^a^	0.2 ± 0.01^a^

^a,^^b,c^Differences between groups indicated with different letters in the same column are significant (*P* < 0.05)

Since those with an insulin resistance HOMA-IR score of ≥ 2.5 were considered positive for insulin resistance, the HOMA-IR score of < 2.5 in groups 1 and 3 and the HOMA-IR score of ≥ 2.5 in groups 2, 4, and 5 without insulin resistance and insulin resistance were determined.

It was shown that group 2 with diabetes had higher serum urea levels than the control group (group 1) (*P* > 0.05), and that this difference was statistically significant in group 4 (*P* < 0.05). Insulin change in serum is presented in Table 3.

There was a significant positive correlation between AST and ALT, TC, Glc, ALB/GLO, and urea (*r* = 0.67**, *r* = 0.66**, *r* = 0.65**, *r* = 412**, *r* = 0.4) (*P* < 0.01). It was determined that there was a significant positive correlation (*r* = 534**, *r* = 0.65**) between ALT and TC enzyme activities, and Glc (*P* < 0.01) (Table 6).

**Table 6:** Correlation relationship between groups

	AST	ALT	TC	Glc	UA	TP	ALB	GLO	ALB/GLO	Urea	Creatinine
AST	1	0.67**	0.66**	0.65**	0.20	− 0.54**	0.03	− 0.46**	0.41**	0.4**	0.006
ALT		1	534**	0.65**	0.17	− 0.45**	− 0.05	− 0.33*	0.28	0.31	0.06
TC			1	0.46**	0.08	− 0.51**	0.34*	− 0.62**	0.57**	− 0.05	− 0.17
Glc				1	0.29	− 0.58**	− 0.24	− 0.33*	0.17	0.27	0.15
UA					1	0.04	− 0.12	0.1	− 0.13	0.2	0.46**
TP						1	0.01	0.81**	− 0.53**	− 0.07	− 0.10
ALB							1	− 0.57**	0.79**	0.69**	− 0.51**
GLO								1	− 0.9**	0.34*	0.21
ALB/GLO									1	− 0.43**	− 0.3
Urea										1	0.44**
Creatinine											1

*Correlation is significant at the 0.5 level (2-tailed); **Correlation is significant at the 0.01 level (2-tailed)

A significant positive correlation (*r* = 0.46**) was found between TC and Glc (*P* < 0.05). It was determined that there was a significant positive correlation (*r* = 0.46**) between UA and creatinine (*P* < 0.05). A significant positive correlation (*r* = 0.81**) was found between TP and GLO (*P* < 0.05). It was determined that there was a significant positive correlation (*r* = 0.79**) between ALB and ALB/GLO (*P* < 0.05). It was determined that there was a significant positive correlation (*r* = 0.34*) between GLO and urea (*P* < 0.05). It was found that there was a significant positive correlation (*r* = 0.44**) between the of urea and creatinine (*P* < 0.05) (Table 6).

### Total *beta* amyloid (Aβ), tau protein, and insulin levels in serum

Aβ protein level in serum was determined as 561.8 ± 23.3, 599.9 ± 18.2, 473.3 ± 34.6, 522.8 ± 6.4, and 489.4 ± 8.8 (ng/L) in groups 1, 2, 3, 4, and 5, respectively. It was determined that groups 3 and 5 were significantly lower than the control group (group 1) (*P* < 0.05). It was determined that group 2 was higher than the control group (group 1) (*P* > 0.05) (Table 7).

**Table 7:** Beta amyloid (Aβ), tau protein, and insulin levels in serum

	Aβ (ng/L)	Tau (ng/L)	Insulin (mU/L)
Group 1	561.8 ± 23.3^ab^	236.6 ± 9.3^a^	9.1 ± 0.3^ac^
Group 2	599.9 ± 18.2^b^	288.8 ± 13.5^b^	8.1 ± 0.2^b^
Group 3	473.3 ± 3.6^c^	171.6 ± 11.2^c^	8.9 ± 0.1^b^
Group 4	522.8 ± 6.4^ac^	165.7 ± 10.7^c^	8.9 ± 0.1^a^
Group 5	489.4 ± 8.8^c^	158.85 ± 7^c^	8.2 ± 0.7^bc^

^a,^^b,c^Differences between groups indicated with different letters in the same column are significant (*P* < 0.05)

Tau protein level was found to be significantly higher in group 2 than the other groups (*P* < 0.05), while groups 3, 4, and 5, which were administered curcumin, were lower than the control and diabetes groups (groups 1 and 2) (*P* < 0.05).

Insulin levels in serum were determined as 9.16 ± 0.36, 8.13 ± 0.2, 8.9 ± 0.1, 8.9 ± 0.1, and 8.2 ± 0.7 (mU/L) in groups 1, 2, 3, 4, and 5, respectively. Groups 2 and 3 (*P* < 0.05) and groups 4 and 5 (*P* > 0.05) were found to be lower than the control group (group 1).

### Aβ protein, Tau protein, and insulin levels in the brain

In the experimental groups, Aβ protein, Tau protein, and insulin levels in group 1 (control), group 2 (diabetes), group 3 (curcumin), group 4 (diabetes—curcumin), and group 5 (curcumin—diabetes) in brain tissue are presented in Table 8.

**Table 8:** Beta amyloid (Aβ), tau protein, and insulin levels in brain tissue

	Aβ (µg/g tissue)	Tau (ng/g tissue)	Insulin (mU/g tissue)
Group 1	1.3 ± 0.04^ab^	131.1 ± 11^a^	43.2 ± 0.7
Group 2	2 ± 0.2^c^	190.9 ± 7^b^	40.5 ± 1.3
Group 3	1.1 ± 0.01^a^	120.2 ± 7.3^a^	40.9 ± 0.7
Group 4	1.6 ± 0.04^bc^	143.9 ± 7.4^a^	40.6 ± 1
Group 5	1.4 ± 0.4^ab^	133.7 ± 7.9^a^	40.4 ± 0.4

^a,^^b,c^Differences between groups indicated with different letters in the same column are significant (*P* < 0.05)

Aβ protein level in brain tissue group 5 was found to be at the same level as the control group (group 1) (*P* > 0.05). It was determined that group 2 was significantly higher than group 4 (*P* < 0.05).

Tau protein level in brain tissue was found to be significantly higher in group 2 compared to the control group (group 1) (*P* < 0.05).

Insulin levels in brain tissue were found to be lower in groups 2, 3, 4, and 5 compared to the control group (group 1) (*P* > 0.05).

### TAS, TOS, and OSI levels in serum

The levels of total antioxidant capacity (STAS), total oxidant capacity (STOS), and OSI (SOSI) in the serum obtained from blood samples were determined in the experimental groups (groups 1, 2, 3, 4, and 5) at the end of the trial. It was determined that the TAS level of group 4 was higher than group 2 (*P* < 0.05) and close to the control group (group 1) (*P* > 0.005). TAS level of group 5 was found to be higher than the control group (group 1) (*P* < 0.05) (Table 9).

**Table 9:** TAS, TOS, and OSI levels in serum

	TAS mmol/L	TOS µmol/L	OSI
Group 1	0.69 ± 0.05^ab^	6.44 ± 1.61^ab^	9.38
Group 2	0.61 ± 0.05^b^	12.3 ± 4.9^b^	23.93
Group 3	0.88 ± 0.8^ac^	2.56 ± 0.2^a^	3.4
Group 4	0.71 ± 0.05^ab^	7.14 ± 1.2^ab^	11.45
Group 5	1.03 ± 0.03^c^	9.16 ± 1.4^ab^	10.77

^a,^^b,c^Differences between groups indicated with different letters in the same column are significant (*P* < 0.05)

TOS level in serum was found to be significantly higher than groups 2 and 5 and the control group (group 1) (*P* < 0.05). It was determined that group 2 was significantly higher than group 4 (*P* < 0.05). It was determined that group 3 was significantly lower than the control group (group 1) (*P* > 0.05).

### TAS, TOS, and OSI levels in brain tissue

The levels of total antioxidant capacity (BTAS), total oxidant capacity (BTOS), and OSI (BOSI) levels in brain tissue are presented in the table (Table 10). TAS levels in brain tissue were found to be significantly lower in groups 2, 4, and 5 compared to the control group (group 1) (*P* > 0.05). Group 3 was found to be significantly higher than the control group (group 1) (*P* < 0.05).

**Table 10:** TAS, TOS, and OSI levels in brain tissue

	TAS mmol/g tissue	TOS µmol/g tissue	OSI
Group 1	2.12 ± 0.58	19.47 ± 6.3^ab^	8.6^ab^
Group 2	1.5 ± 0.47	33.19 ± 5.9^ac^	20.46^ab^
Group 3	2.68 ± 0.57	13.27 ± 2.9^b^	6.55^a^
Group 4	1.90 ± 0.49	22.84 ± 4.6^ab^	23.34^b^
Group 5	1.8 ± 0.64	49.79 ± 8.5^c^	20.58^ab^

^a,^^b,c^Differences between groups indicated with different letters in the same column are significant (*P* < 0.05)

TOS level in brain tissue was found to be higher in groups 2 and 4 than the control group (group 1) (*P* < 0.05). It was determined that group 3 was significantly lower than the control group (group 1) (*P* > 0.05). Group 5 was found to be significantly higher than the control group (group 1) (*P* < 0.05).

It was determined that there was a positive correlation between glucose and BTOS, STOS, BOSI, and SOSI (*r* = 0.33*, *r* = 0.35*, *r* = 0.34*, *r* = 0.33*) (*P* < 0.5). It was determined that there was a positive correlation (*r* = 0.34*) between BTOS and SOSI (*P* < 0.5). It was determined that there was a significant positive correlation (*r* = 0.96**) between STOS and SOSI (*P* < 0.05). Correlation between groups is presented (Table 11).

**Table 11:** The correlation relationship between TAS, TOS, and OSI in all brain tissues (BOSI, BTAS, and BTOS) and serum (Glc, SOSI, STAS, and STOS) among the groups

	Glc	BTAS	STAS	BTOS	STOS	BOSI	SOSI
Glc	1	− 0.18	− 0.05	0.33*	0.35*	0.34*	0.33*
BTAS		1	0.01	− 0.18	0.08	− 0.51**	− 0.009
STAS			1	0.33	− 0.16	− 0.03	− 0.34*
BTOS				1	0.03	− 0.16	0.34*
STOS					1		0.96**
BOSI						1	0.18
SOSI							1

*Correlation is significant at the 0.5 level (2-tailed); **Correlation is significant at the 0.01 level (2-tailed)

## Discussion

Diabetes mellitus is a chronic metabolic disease with a worldwide increasing prevalence (Guariguata et al. 2014). Curcumin is a natural polyphenol with various pharmacological activities such as anti-inflammation, anti-cancer, and anti-oxidation (Hussain et al. 2017). Its pharmacological effects are extensively involving in the modulation of inflammatory molecules, transcription factors, enzymes, protein kinases, growth factors, cell cycle regulatory proteins, metal ions, DNA, lipids, and proteins, among other areas. Animal studies have suggested that curcumin could serve as a therapeutic option for a broad spectrum of human diseases including diabetes, obesity, cancer, and systemic chronic diseases as well as psychiatric and neurological disorders. These studies point out the therapeutic potential of curcumin against neurodegenerative diseases (Reddy et al. 2016). Alzheimer’s disease is one of the most important complications of diabetes and is the most common type of dementia in the elderly population (Rocchi et al. 2003). AD is characterized by progressive decline in cognitive functions and accumulation of Aβ that forms senile plaques in the brain (Price et al. 1995). The development of nanocarriers for the delivery of curcumin to Alzheimer’s disease (AD) has been the subject of significant research in recent years. However, it remains uncertain whether these formulations are effective, safe, and suitable for use (Ahmad et al. 2024). New approaches suggest that cognitive deficits may arise from abnormalities in multiple signaling pathways, particularly those mediated by inflammatory and oxidative stress. Thus, multi-target compounds can effectively combat cognitive deficits. Since curcumin interacts with numerous molecules involved in these pathways, it has been indicated as a promising compound for the treatment/prevention of cognitive decline (Voulgaropoulou et al. 2019). At the beginning of the study, the mean body weight of the rats was approximately 265 ± 10 g. At the end of the study, it was determined that the body weight gain in groups 2 and 4, in which experimental diabetes was induced by STZ, was significantly lower than the control group (group 1) (*P* > 0.05), while it decreased slightly in the other groups (*P* > 0.05). An increase in body weight was observed when curcumin (100 mg/kg) was administered to rats that developed diabetes by administering low doses of streptozotocin along with a high-energy diet for 8 weeks (Yang et al. 2018).

In our study, it was determined that there was a 14.13% decrease in live weight in group 4. In the study by Kuhad and Chopra (2007), male Wistar albino rats were given 65 mg/kg STZ to induce diabetes, while 60 mg/kg was given to the diabetes curcumin group by oral gavage for 10 weeks for treatment. At the end of the study, it was observed that there was an 8.1% decrease in body weight in the diabetes curcumin group, and an 11.40% increase in body weight in the curcumin group at the end of the study. In our study, there was a 5% increase in body weight in group 3. In another study, STZ (60 mg/kg) was given to male rats, and diabetes was formed, and curcumin (150 mg/kg/day) was given by oral gavage for 12 weeks for treatment; and the control group, the diabetic group, and the diabetes group were additionally administered. The report suggests that the diabetic group treated with curcumin exhibited a higher live weight gain compared to the untreated diabetic group (Huang et al. 2013). On the 35th day, it was observed that the maximum increase in body weight occurred in the control group (group 1), while the minimum increase was observed in group 4 (*P* < 0.05).

There are studies showing that curcumin administration to diabetic rats regulates blood glucose levels (Nishiyama et al. 2005; Huang et al. 2013; Mustafa 2016; Rahimi et al. 2016). In a study conducted in parallel with our study, 3-month-old male rats were made diabetic by STZ (35 mg/kg), and diabetes group was given curcumin (100 mg/kg) for 8 weeks by oral gavage, and diabetes group was given at the end of the study. No significant difference was found between diabetic groups in blood glucose level (Suryanarayana et al. 2005). Sprague–Dawley male rats were made diabetic by administered STZ (55 mg/kg) and rats fed with 200 mg/kg curcumin. At the end of the study, group diabetic and diabetes curcumin group, blood glucose levels were determined as 498 (mg/dl) and 478 (mg/dl), respectively, and it was reported that the effect of curcumin on reducing hyperglycemia was not significant (Majithiya and Balaraman 2005). In another study, Sprague Dawley rats rendered diabetic with STZ (45 mg/kg) were administered curcumin 200 mg/kg/day by oral gavage for 14 days, and it was reported that the effect of curcumin administration on serum glucose concentration was not significant at the end of the study (Nishizono et al. 2000). Our study results indicate that at the end of the experiment, the blood glucose levels of groups 2, 4, and 5 with diabetes were significantly elevated compared to the control group (group 1). Additionally, although not statistically significant, there was a slight decrease in the blood glucose levels of the diabetic groups receiving curcumin treatment.

Alanine aminotransferase (ALT) and aspartate aminotransferase (AST) are liver enzymes commonly used for liver function tests. At the end of the study, it was determined that ALT and AST enzyme activity increased in the serum of (groups 2, 4, and 5) experimental diabetic rats. These results are supported by studies showing that DM is associated with elevated hepatic enzyme activity in serum, which may be a result of liver cell destruction or changes in membrane permeability indicating severe hepatocellular damage. In a study, 60 mg/kg curcumin was administered to adult male Wistar albino rats for whom diabetes was induced with STZ (60 mg/kg) for 30 days by oral gavage, and it was reported that the effect of curcumin on serum AST level was not significant, but it significantly decreased ALT level (Palma et al. 2014). In another study, diabetes was induced by STZ (50 mg/kg) injection in rats, and curcumin was given 80 mg/kg/day by oral gavage for 60 days. It was reported that ALT and AST enzyme activity decreased significantly (Mustafa 2016). In our study, it was observed that curcumin administration had a positive effect on reducing the liver enzymes—ALT and AST—in serum of experimental diabetes-induced rats, which was consistent with the literature.

Hyperlipidemia is a complication of DM. Significant changes in the lipid profile can be seen depending on the induction of DM. In a study, after inducing diabetes with STZ (65 mg/kg) in rats, curcumin was administered at a dose of 10 mg/kg for 45 days. It has been reported that the water-soluble form of curcumin lowers TC and improves the lipid profile (Abdel Aziz et al. 2012). In another study, DM was induced by intravenous injection of 40 mg/kg STZ in rats. The rats were given 90 mg/kg curcumin with yogurt at 1.0 mL per day per rat by oral gavage for 35 days. At the end of the study, it was reported that TC levels were 21% higher in the diabetes group and 22% higher in the diabetes curcumin group compared to the control group (Gutierres et al. 2012). In another study, 120 mg/kg of alloxan monohydrate was administered intraperitoneally to Sprague Dawley rats to induce diabetes. Rats were administered 200 mg/kg of turmeric extract by oral gavage for 56 days. At the end of the study, it was reported that the TC levels were 24% higher in the diabetes group compared to the control group, and it approached the control group in the group in which diabetes was formed and turmeric extract was given (El-Hadary and Sitohy 2021). In our study, it was determined that the TC level was higher in the diabetic groups (groups 2, 4, and 5) compared to the control group.

It was suggested that urea, creatinine, and uric acid levels, which are indicators of kidney function parameters, were affected in rats with diabetes, and that the administration of curcumin derivatives to diabetic rats may protect kidney function (Wu et al. 2014; Xu et al. 2018; El-Hadary and Sitohy 2021). In a study investigating the effect of stevia and turmeric extracts on hypoglycemia in rats, it was reported that turmeric extracts significantly decreased the elevated urea, creatinine, and uric acid levels in rats with diabetes and had a protective effect on kidney functions (El-Hadary and Sitohy 2021). In our study, there was an increase in urea and creatinine levels in the groups with diabetes (groups 2 and 4) compared to the control (group 1) group, but this increase was not statistically significant. In the group with diabetes on the 28th day (group 5), it was determined that the urea and creatinine levels were not affected and were close to the control group. It was observed that the uric acid level was increased in groups 2 and 5 compared to the control group (*P* > 0.05) in the diabetes-induced groups, while this increase was the highest in group 4 (*P* < 0.05). In another study, 15 and 30 mg/kg curcumin were administered to diabetic rats induced by 65 mg/kg STZ for 2 weeks, and at the end of the study, polyuria, increased urinary albumin excretion, increased serum creatinine, and increased blood urea nitrogen were detected in diabetic rats. It was reported that as a result of curcumin treatment of 15 and 30 mg/kg per day in diabetic rats, it effectively reduced diabetic proteinuria, polyuria, and increased serum creatinine and blood urea nitrogen. It was reported that creatinine and urea clearance were also significantly reduced following the administration of curcumin to diabetic rats compared to untreated diabetic rats, and it had a healing effect on renal dysfunction by reducing oxidative stress (Sharma et al. 2006). In our study, urea and creatinine levels were higher in groups 2 and 4 compared to the control (group 1) group, and group 5 approached the control group.

In one study, diabetes was induced by injection of STZ (50 mg/kg) in rats, and curcumin (80 mg/kg/day) was given by oral gavage for 60 days. At the end of this study, there was a significant decrease in albumin and total protein levels in the diabetic group compared to the control group, and a significant increase in these parameters in the diabetic group treated with curcumin (Mustafa 2016). In another study, experimental T2DM was induced in adult rats and treated with curcumin (80 mg/kg) by oral gavage for 8 weeks. At the end of the study, the total protein level decreased significantly in the diabetes group compared to the control group, and there was a significant increase in the curcumin-treated diabetes group (Al-Saud 2020). In our study, the total protein level among the groups decreased in the diabetes-induced groups compared to the control group (*P* > 0.05), and this decrease was more pronounced in group 5 (*P* < 0.05). Among the groups, group 5 had the highest amount of albumin and the lowest amount of globulin (*P* < 0.05).

Insulin resistance is a condition where insulin-dependent glucose update by cells is impaired. Insulin resistance is also an important risk factor for related chronic diseases such as type 2 diabetes, atherosclerosis, and cardiovascular disease. Insulin resistance is an important component of the metabolic syndrome, which consists of a number of risk factors such as abdominal obesity, hypertension, and dyslipidemia (Hekmatdoost et al. 2011). Regarding the pathophysiology of diabetes, there are studies investigating the therapeutic effect of curcumin in cases of insulin action, insulin secretion malfunction, β-cell dysfunction, insulin secretion reductions, and insulin resistance (Kim et al. 2016; Zheng et al. 2018; Lee et al. 2020; Al-Saud 2020). In a study, after induction of experimental T2DM in adult rats, curcumin (80 mg/kg) was administered by oral gavage for 8 weeks, and it was reported that glucose level and insulin resistance (HOMA-IR) decreased after curcumin treatment in diabetes groups at the end of the study (Al-Saud 2020). In another study, high-fat diet-induced diabetic Sprague Dawley rats were given 80 mg/kg curcumin for 15 days, and it was reported that curcumin exhibited an anti-hyperglycemic effect and improved insulin sensitivity at the end of the study (El-Moselhy et al. 2011). An experimental T2DM model was established in Otsuka-Long-Evans-Tokushima Fatty (OLETF) rats and treated for 40 weeks by administering curcumin (100 mg/kg). At the end of the study, it was reported that there was no difference in HOMA-IR values between the control, diabetes, and diabetes curcumin groups (Kim et al. 2016). In our study, it was observed that in the diabetic groups (groups 2, 4, and 5), the insulin resistance score, HOMA-IR, was ≥ 2.5, while in the non-diabetic groups (groups 1 and 3), HOMA-IR was < 2.5. Alzheimer’s disease (AD) is a progressive, neurodegenerative disease characterized by extracellular amyloid beta (Aβ) protein deposits and intracellular neurofibrillary tangles of hyperphosphorylated tau protein (Patil et al. 2013). Curcumin is a component of turmeric, a spice used in many types of cooking. Epidemiological evidence shows that populations consuming foods containing significant amounts of curcumin have a lower risk of Alzheimer’s disease (AD). These results suggest that this compound may have a neuroprotective effect. Curcumin also protects against toxicity when β-amyloid is applied to produce animal models of AD, and curcumin reduces the formation of β-amyloid from amyloid precursor protein. It has been reported that it inhibits the aggregation of β-amyloid in layers. The neuroprotective effect of curcumin was reported in a study in transgenic mice with β-amyloid production (Potter 2013). Due to its lipophilic properties, curcumin crosses the blood–brain barrier (BBB) and binds to amyloid deposits. The binding of curcumin to Aβ has been shown to divert the aggregation pathway toward the formation of non-toxic aggregates, indicating a change in aggregation equilibrium (Rao et al. 2015). Various in vivo studies have revealed that curcumin supports the breakdown of existing amyloid deposits and prevents the accumulation of new amyloid deposits, even reducing the size of remaining deposits (Lim et al. 2001; Garcia-Alloza et al. 2007; Liu et al. 2010; Zhang et al. 2010). In the conducted study, the application of curcumin to STZ-induced diabetic rats significantly improved blood sugar levels, redox status, and cellular stress. As a result, curcumin was indicated as a potential neuroprotective agent against diabetes-induced hippocampal neurodegeneration (Keshk et al. 2020). At the end of our study, it was observed that there was a significant difference between groups 2 and 4 in terms of brain β-amyloid protein levels, and the decrease was higher in the group given curcumin. After feeding a high-fat diet for 8 weeks, rats induced T2DM by 35 mg/kg STZ were given curcumin and curcumin nanoparticles (CURNP) by oral gavage for 6 weeks. At the end of the study, it was found that serum insulin levels, as well as brain and hippocampus Aβ-42 protein and tau protein levels, were significantly higher in the diabetes group compared to the control group. In conclusion, the findings of this study demonstrate that curcumin inhibits amyloidogenesis and the hyperphosphorylation of tau proteins in the brain hippocampus of rats (Abdulmalek et al. 2021). At the end of our study, the levels of Aβ and tau protein in the serum and brain tissue of group 2 were found to be higher than the control group, and treatment with curcumin in group 5 has a positive effect on the reduction.

Antioxidants play an important role in the prevention and improvement of diabetic complications (Rahimi et al. 2005). Curcumin, a powerful antioxidant, protects neurons from oxidative damage by regulating mitochondrial membrane potential and inhibiting intracellular ROS production. Accordingly, many studies have shown that curcumin reduces diabetes and diabetes-related symptoms (Aggarwal and Harikumar 2009; Acar et al. 2012). In the study investigating the effect of STZ-induced diabetic rats on the oxidant/antioxidant balance in the sciatic nerve and brain tissue, it was observed that TOS and OSI levels in the brain tissue of diabetic rats increased significantly compared to control rats, and TAS levels decreased significantly compared to the control. It was reported that TOS and OSI levels in brain tissue decreased in diabetic rats treated with curcumin while TAS level increased, and curcumin has the ability to reduce oxidative damage in the brain tissue of diabetic rats (Acar et al. 2012). In our study, it was determined that the level of TOS increased in groups 2 and 5 with diabetes compared to the control group (group 1), and the level of TAS activity in this tissue decreased significantly compared to the control group. It was determined that TOS level in the brain tissue was highest in group 2 with diabetes while TOS level decreased and TAS level increased in the curcumin treatment group 4.

In one study, diabetes was induced in rats with 55 mg/kg of STZ, and 50 to 150 mg/kg of curcumin was given to the groups by oral gavage for 42 days to treat it. The activities of enzymes involved in the antioxidant defense system were investigated. At the end of this study, although the TAS level of diabetic rats did not change with curcumin treatment, a significant increase was observed in TOS, OSI, and MDA levels in diabetic rats group. At the end of the treatment of diabetic rats with curcumin, it was observed that the oxidative stress parameters returned to the normal range. At the end of the study, it was reported that curcumin exhibits potent antioxidant properties and therefore may be beneficial in ameliorating the oxidative stress caused by diabetes and improving the cardiac damage caused by diabetes in the heart tissue (Naghdi et al. 2022). In our study, it was determined that the TAS level in the serum was lower in group 2 compared to the control group (group 1), and group 4 returned to normal levels with curcumin treatment. It was predicted that TOS activities in the serum were significantly higher in group 2 with diabetes compared to the control (group 1) group; and in group 4, it approached the control group with curcumin treatment, and it was predicted that curcumin could improve the complications of diabetes by decreasing the oxidative stress caused by diabetes and increasing the antioxidant activity.

In a study conducted in parallel with our study, diabetes was induced by injection of 50 mg/kg STZ in adult rats and treated with oral curcumin (100 mg/kg) administration to rats for 42 days. At the end of the study, it was reported that curcumin reduced testicular damage by reducing oxidative stress in diabetic rats (Kanter et al. 2013). In another study, it was reported that the application of curcumin and turmeric partially improved oxidative stress in STZ-induced diabetic rats. It has been reported that the administration of curcumin to STZ-induced diabetic rats has an inhibitory effect on oxidative stress by modulating antioxidant enzyme activities and lipid peroxidation (Palma et al. 2014). In a study investigating the role of curcumin in the pancreatic tissue of diabetes through cellular stress induced by STZ in Wistar albino male rats, it was shown that curcumin can reduce oxidative stress, ER stress, and related inflammation and protect pancreatic beta cells from apoptotic damage under hyperglycemic conditions (Rashid and Sil 2015). In another study investigating oxidative damage and mitochondrial dysfunction in diabetic rat brain, curcuminoids showed protective effects against oxidative damage and mitochondrial dysfunction in diabetic rat brain (Rastogi et al. 2008). In our study, the level of brain tissue and blood serum TOS activity was higher in group 2 with diabetes compared to the control (group 1) group, and significantly decreased in group 4 with curcumin treatment. The level of TAS activity in serum and brain tissue decreased in group 2 compared to the control (group 1) group, and approached the control group in group 4. Therefore, curcumin has an effect of reducing oxidative stress, which increases with diabetes, and increasing antioxidant activity.

In summary, curcumin can improve the complications of diabetes by increasing the antioxidant activity and reducing oxidative stress caused by diabetes. Curcumin may be recommended as a supportive treatment in addition to standard diabetes treatment. However, experimental studies with longer trial periods are needed to better demonstrate the efficacy of curcumin.

## Data Availability

No datasets were generated or analysed during the current study.

## Abbreviations

AD: Alzheimer’s disease
Alb: Albumin
ALT: Alanine aminotransferase
AST: Aspartate transaminase
Aβ: Amyloid beta
BOSI: Oxidative stress index in all brain tissues
BTAS: Total antioxidant capacity in all brain tissues
BTOS: Total oxidant capacity in all brain tissues
DM: Diabetes mellitus
ELISA: Enzyme-linked immunosorbent assay
Glc: Glucose
Glo: Globulin
HOMA-IR: Insulin resistance
i.p.: Intraperitoneally
OSI: Oxidative stress index
PBS: Phosphate buffered saline
SOSI: Oxidative stress index in serum
STAS: Total antioxidant capacity in serum
STOS: Total oxidant capacity in serum
STZ: Streptozotocin
TAS: Total antioxidant capacity
TC: Total cholesterol
TOS: Total oxidant capacity
TP: Total protein
UA: Uric acid

## References

[CR1] Abdel Aziz MT, El-Asmar MF, El-Ibrashy IN et al (2012) Effect of novel water soluble curcumin derivative on experimental type-1 diabetes mellitus. Diabetol Metab Syndr 4(1):30 22762693 10.1186/1758-5996-4-30 PMC3533893

[CR2] Abdulmalek S, Nasef M, Awad D et al (2021) Protective effect of natural antioxidant, curcumin nanoparticles, and zinc oxide nanoparticles against type 2 diabetes-promoted hippocampal neurotoxicity in rats. Pharmaceutics 13(11):19–37 10.3390/pharmaceutics13111937 PMC8621156 34834352

[CR3] Acar A, Akil E, Alp H et al (2012) Oxidative damage is ameliorated by curcumin treatment in brain and sciatic nerve of diabetic rats. Int J Neurosci 122(7):367–372 22248035 10.3109/00207454.2012.657380

[CR4] Aggarwal BB, Harikumar KB (2009) Potential therapeutic effects of curcumin, the anti-inflammatory agent, against neurodegenerative, cardiovascular, pulmonary, metabolic, autoimmune and neoplastic diseases. Int J Biochem Cell Biol 41(1):40–59 18662800 10.1016/j.biocel.2008.06.010 PMC2637808

[CR5] Ahmad F, Karan A, Sharma R et al (2024) Evolving therapeutic ınterventions for the management and treatment of Alzheimer’s disease. Ageing Res Rev 95:102229 38364913 10.1016/j.arr.2024.102229

[CR6] Ali MM, Agha FG (2009) Amelioration of streptozotocin induced diabetes mellitus, oxidative stress and dyslipidemia in rats by tomato extract lycopene. Scand J Clin Lab 69(3):371–379 10.1080/00365510802658473 19148834

[CR7] Al-Saud NBS (2020) Impact of curcumin treatment on diabetic albino rats. Saudi J Biol Sci 27(2):689–694 32210689 10.1016/j.sjbs.2019.11.037 PMC6997849

[CR8] Barbour HM, Davisdon W (1988) Studies on measurement of plasma magnesium: application of the Magon dye method to the “Monarch” centrifugal analyzer. Clin Chem 34(10):2103–2105 3168223

[CR9] Baynes JW (1991) Role of oxidative stress in development of complications in diabetes. Diabetes 40:405–412. 10.2337/diab.40.4.405 2010041 10.2337/diab.40.4.405

[CR10] Bonora E, Targher G, Alberiche M et al (2000) Homeostasis model assessment closely mirrors the glucose clamp technique in the assessment of insulin sensitivity: studies in subjects with various degrees of glucose tolerance and insulin sensitivity. Diabet Care 23:57–63. 10.2337/diacare.23.1.57 10.2337/diacare.23.1.57 10857969

[CR11] Chao AC, Lee TC, Juo SH et al (2016) Hyperglycemia increases the production of amyloid beta-peptide leading to decreased endothelial tight junction. CNS Neurosci Ther 22:291–297 26842741 10.1111/cns.12503 PMC6492831

[CR12] El-Hadary A, Sitohy M (2021) Safely effective hypoglycemic action of stevia and turmeric extracts on diabetic Albino rats. J Food Biochem 45(1):13549 10.1111/jfbc.13549 33161596

[CR13] El-Moselhy MA, Taye A, Sharkawi SS et al (2011) The antihyperglycemic effect of curcumin in high fat diet fed rats. Role of TNF-alpha and free fatty acids. Food Chem Toxicol 49(5):1129–1140 21310207 10.1016/j.fct.2011.02.004

[CR14] Erel O (2004) A new automated direct measurement method for total antioxidant capacity using a new generation, more stable ABTS radical cation. Clin Biochem 37:277–285 15003729 10.1016/j.clinbiochem.2003.11.015

[CR15] Erel O (2005) A new automated colorimetric method for measuring total oxidant status. Clin Biochem 38:1103–1111 16214125 10.1016/j.clinbiochem.2005.08.008

[CR16] Farooqui T, Farooqui AA (2019) Curcumin: historical background, chemistry, pharmacological action, and potential therapeutic value. Curcumin Neurol Psychiatr Disord 23–44

[CR17] Garcia-Alloza M, Borrelli LA, Rozkalne A et al (2007) Curcumin labels amyloid pathology in vivo, disrupts existing plaques, and partially restores distorted neurites in an Alzheimer mouse model. J Neurochem 102(4):1095–1104 17472706 10.1111/j.1471-4159.2007.04613.x

[CR18] Guariguata L, Whiting DR, Hambleton I, Beagley, et al (2014) Global estimates of diabetes prevalence for 2013 and projections for 2035. Diabetes Res Clin Pract 103(2):137–149 24630390 10.1016/j.diabres.2013.11.002

[CR19] Gutierres VO, Pinheiro CM, Assis RP et al (2012) Curcumin-supplemented yoghurt improves physiological and biochemical markers of experimental diabetes. Br J Nutr 108(4):440–448 22067670 10.1017/S0007114511005769

[CR20] Hamze R, Delangre E, Tolu S et al (2022) Type 2 diabetes mellitus and Alzheimer’s disease: shared molecular mechanisms and potential common therapeutic targets. Int J Mol Sci 23(23):15287 36499613 10.3390/ijms232315287 PMC9739879

[CR21] Hekmatdoost A, Hosseini-Esfahani MP, F, et al (2011) Dietary fatty acid composition and metabolic syndrome in Tehranian adults. Int J Appl Basic Nutr Sci 27(10):1002–1007 10.1016/j.nut.2010.11.004 21907897

[CR22] Huang J, Huang K, Lan T et al (2013) Curcumin ameliorates diabetic nephropathy by inhibiting the activation of the SphK1-S1P signaling pathway. Mol Cell Endocrinol 365(2):231–240 23127801 10.1016/j.mce.2012.10.024

[CR23] Hussain Z, Thu HE, Amjad MW et al (2017) Exploring recent developments to improve antioxidant, anti-inflammatory and antimicrobial efficacy of curcumin: a review of new trends and future perspectives. Mater Sci Eng C-Mater Biol Appl 77(1):1316–1326 28532009 10.1016/j.msec.2017.03.226

[CR24] Kanter M, Aktas C, Erboga M (2013) Curcumin attenuates testicular damage, apoptotic germ cell death, and oxidative stress in streptozotocin-induced diabetic rats. Mol Nutr Food Res 57(9):1578–1585 22930655 10.1002/mnfr.201200170

[CR25] Keshk WA, Elseady WS, Sarhan NI et al (2020) Curcumin attenuates cytoplasmic/endoplasmic reticulum stress, apoptosis and cholinergic dysfunction in diabetic rat hippocampus. Metab Brain Dis 35:637–647 32172517 10.1007/s11011-020-00551-0

[CR26] Kim BH, Lee ES, Choi R et al (2016) Protective effects of curcumin on renal oxidative stress and lipid metabolism in a rat model of type 2 diabetic nephropathy. Yonsei Med J 57(3):664–673 26996567 10.3349/ymj.2016.57.3.664 PMC4800357

[CR27] Kuhad A, Chopra K (2007) Curcumin attenuates diabetic encephalopathy in rats: behavioral and biochemical evidences. Eur J Pharmacol 576(1–3):34–42 17822693 10.1016/j.ejphar.2007.08.001

[CR28] Kurkinen M, Fulek M, Fulek K et al (2023) The amyloid cascade hypothesis in Alzheimer’s disease: should we change our thinking? Biomolecules 13(3):453 36979388 10.3390/biom13030453 PMC10046826

[CR29] Lee HJ, Seo HI, Cha HY et al (2018) Diabetes and Alzheimer’s disease: mechanisms and nutritional aspects. Clin Nutr Res 7(4):229–240 30406052 10.7762/cnr.2018.7.4.229 PMC6209735

[CR30] Lee ES, Kwon MH, Kim HM et al (2020) Curcumin analog CUR5-8 ameliorates nonalcoholic fatty liver disease in mice with high-fat diet-induced obesity. Metabolism 103:154015 31758951 10.1016/j.metabol.2019.154015

[CR31] Lim GP, Chu T, Yang F et al (2001) The curryspice curcumin reduces oxidative damage and amyloid pathology in an Alzheimer transgenicmouse. J Neurosci 21(21):8370–8377 11606625 10.1523/JNEUROSCI.21-21-08370.2001 PMC6762797

[CR32] Liu H, Li Z, Qiu D et al (2010) The inhibitory effects of different curcuminoids on β-amyloid protein, β-amyloid precursor protein and β-site amyloid precursor protein cleaving enzyme 1 in swAPP HEK293 cells. Neurosci Lett 485(2):83–88 20727383 10.1016/j.neulet.2010.08.035

[CR33] Majithiya JB, Balaraman R (2005) Time-dependent changes in antioxidant enzymes and vascular reactivity of aorta in streptozotocin-induced diabetic rats treated with curcumin. J Cardiovasc Pharmacol 46(5):697–705 16220078 10.1097/01.fjc.0000183720.85014.24

[CR34] Mishra D, Flora SJS (2008) Differential oxidative stress and DNA damage in rat brain regions and blood following chronic arsenic exposure. Sage J 24(4):247–256 10.1177/0748233708093355 19022878

[CR35] Mustafa HN (2016) The role of curcumin in streptozotocin-induced hepatic damage and the trans-differentiation of hepatic stellate cells. Tissue Cell 48(2):81–88 26905192 10.1016/j.tice.2016.02.003

[CR36] Naghdi A, Goodarzi MT, Karimi J et al (2022) Effects of curcumin and metformin on oxidative stress and apoptosis in heart tissue of type 1 diabetic rats. J Cardiovasküler Thorac Res 14(2):128–137 10.34172/jcvtr.2022.23 PMC9339728 35935389

[CR37] Nishiyama T, Mae T, Kishida H et al (2005) Curcuminoids and sesquiterpenoids in turmeric (Curcuma longaL.) suppress an ıncrease in blood glucose level in type 2 diabetic KK-Ay mice. J Agric Food Chem 53(4):959–963 15713005 10.1021/jf0483873

[CR38] Nishizono S, Hayami T, Ikeda I et al (2000) Protection against the diabetogenic effect of feeding tert-butylhydroquinone to rats prior to the administration of streptozotocin. Biosci Biotechnol Biochem 64(6):1153–1158 10923784 10.1271/bbb.64.1153

[CR39] Palma HE, Wolkmer P, Gallio M et al (2014) Oxidative stress parameters in blood, liver, and kidney of diabetic rats treated with curcumin and/or insülin. Mol Cell Biochem 386(1–2):199–210 24130039 10.1007/s11010-013-1858-5

[CR40] Patil SP, Tran N, Geekiyanage H et al (2013) Curcumin-induced upregulation of the anti-tau cochaperone BAG2 in primary rat cortical neurons. Neurosci Lett 554(24):121–125 24035895 10.1016/j.neulet.2013.09.008 PMC3825752

[CR41] Potter PE (2013) Curcumin: a natural substance with potential efficacy in Alzheimer’s disease. J Exp Pharmacol 5:23–31 27186134 10.2147/JEP.S26803 PMC4863538

[CR42] Price DL, Sisodia SS, Gandy SE (1995) Amyloid β amyloidosis in Alzheimer’s disease. Curr Opin Neurol 8(4):268–274 7582041 10.1097/00019052-199508000-00004

[CR43] Rafiee Z, Nejatian M, Daeihamed M et al (2019) Applications of curcumin-loaded nanocarriers for food, pharmaceutical and cosmetic purposes. Trends Food Sci Technol 21:3468–3497 10.1080/10408398.2018.1495174 30001150

[CR44] Rahimi R, Nikfar S, Larijani B et al (2005) A review on the role of antioxidants in the management of diabetes and its complications. Biomed Pharmacother 59(7):365–373 16081237 10.1016/j.biopha.2005.07.002

[CR45] Rahimi HR, Mohammadpour AH, Dastani M et al (2016) The effect of nano-curcumin on HbA1c, fasting blood glucose and lipid profile in diabetic subjects: a randomized clinical trial. Avicenna J Phytomedicine 6(5):567–577 PMC5052420 27761427

[CR46] Rao PP, Mohamed T, Teckwani K et al (2015) Curcumin binding to beta amyloid: a computational study. Chem Biol Drug Des 86:813–820 25776887 10.1111/cbdd.12552

[CR47] Rashid K, Sil PC (2015) Curcumin enhances recovery of pancreatic islets from cellular stress induced inflammation and apoptosis in diabetic rats. Toxicol Appl Pharmacol 282(3):297–310 25541178 10.1016/j.taap.2014.12.003

[CR48] Rastogi M, Ojha RP, Rajamanickam GV et al (2008) Curcuminoids modulates oxidative damage and mitochondrial dysfunction in diabetic rat brain. Free Radical Res 11–12:999–1005 10.1080/10715760802571988 19031318

[CR49] Reddy PH, Manczak M, Yin X et al (2016) Protective effects of a natural product, curcumin, against amyloid beta induced mitochondrial and synaptic toxicities in Alzheimer’s disease. J Investig Med 64(8):1220–1234 27521081 10.1136/jim-2016-000240 PMC5256118

[CR50] Rocchi A, Pellegrini S, Siciliano G et al (2003) Causative and susceptibility genes for Alzheimer’s disease: a review. Brain Res Bull 61(1):1–24 12788204 10.1016/s0361-9230(03)00067-4

[CR51] Sharma S, Kulkarni SK, Chopra K (2006) Curcumin, the active principle of turmeric (Curcuma longa), ameliorates diabetic nephropathy in rats. Clin Exp Pharmacol Physiol 33(10):940–945 17002671 10.1111/j.1440-1681.2006.04468.x

[CR52] Silveira AC, Dias JP, Santos VM et al (2019) The action of polyphenols in diabetes mellitus and Alzheimer’s disease: a common agent for overlapping pathologies. Curr Neuropharmacol 17(7):590–613 30081787 10.2174/1570159X16666180803162059 PMC6712293

[CR53] Stohs SJ, Chen O, Ray SD et al (2020) Highly bioavailable forms of curcumin and promising avenues for curcumin-based research and application: a review. Molecules 25:1397 32204372 10.3390/molecules25061397 PMC7144558

[CR54] Suryanarayana P, Saraswat M, Mrudula T et al (2005) Curcumin and turmeric delay streptozotocin-induced diabetic cataract in rats. Invest Ophthalmol vis Sci 46(6):2092–2099 15914628 10.1167/iovs.04-1304

[CR55] Voulgaropoulou SD, Van Amelsvoort TAMJ, Prickaerts J et al (2019) The effect of curcumin on cognition in Alzheimer’s disease and healthy aging: a systematic review of pre-clinical and clinical studies. Brain Res 1725:146476 31560864 10.1016/j.brainres.2019.146476

[CR56] Wu W, Geng H, Liu Z et al (2014) Effect of curcumin on rats/mice with diabetic nephropathy: a systematic review and meta-analysis of randomized controlled trials. J Tradit Chin Med 34(4):419–429 25185359 10.1016/s0254-6272(15)30041-8

[CR57] Xu X, Cai Y, Yu Y (2018) Effects of a novel curcumin derivative on the functions of kidney in streptozotocin-induced type 2 diabetic rats. Inflammopharmacology 26(5):1257–1264 29582239 10.1007/s10787-018-0449-1 PMC6153927

[CR58] Yang F, Yu J, Ke F et al (2018) Curcumin alleviates diabetic retinopathy in experimental diabetic rats. Ophthalmic Res 60(1):43–54 29597206 10.1159/000486574

[CR59] Zafar M, Naeem-ul-Hassan NS, Ahmed M et al (2009) Altered liver morphology and enzymes in streptozotocin-induced diabetic rats. Int J Morphol 27:719–725

[CR60] Zhang C, Browne A, Child D et al (2010) Curcumin decreases amyloid-beta peptide levels by attenuating the maturation of amyloid-beta precursor protein. J Biol Chem 285(37):28472–28480 20622013 10.1074/jbc.M110.133520 PMC2937872

[CR61] Zheng J, Cheng J, Zheng S et al (2018) Curcumin, A polyphenolic curcuminoid with ıts protective effects and molecular mechanisms in diabetes and diabetic cardiomyopathy. Front Pharmacol 9(1):472–482 29867479 10.3389/fphar.2018.00472 PMC5954291

